# Evaluation of Bacterial and Fungal Biomarkers for Differentiation and Prognosis of Patients with Inflammatory Bowel Disease

**DOI:** 10.3390/microorganisms11122882

**Published:** 2023-11-29

**Authors:** Hyuk Yoon, Sunghyouk Park, Yu Kyung Jun, Yonghoon Choi, Cheol Min Shin, Young Soo Park, Nayoung Kim, Dong Ho Lee

**Affiliations:** 1 Department of Internal Medicine, Seoul National University Bundang Hospital, Seongnam 13620, Republic of Korea; juk0220@nate.com (Y.K.J.); moonsin12345@naver.com (Y.C.); scm6md@gmail.com (C.M.S.); dkree@snubh.org (Y.S.P.); nayoungkim49@empas.com (N.K.); dhljohn@yahoo.co.kr (D.H.L.); 2 Department of Internal Medicine and Liver Research Institute, Seoul National University College of Medicine, Seoul 03080, Republic of Korea; 3 Department of Manufacturing Pharmacy, Natural Products Research Institute, College of Pharmacy, Seoul National University, Seoul 08826, Republic of Korea; 4 Department of Biological Sciences, College of Natural Sciences, Seoul National University, Seoul 08826, Republic of Korea

**Keywords:** microbiome, mycobiome, inflammatory bowel disease, biomarkers

## Abstract

This study aimed to evaluate bacterial and fungal biomarkers to differentiate patients with inflammatory bowel disease (IBD), predict the IBD prognosis, and determine the relationship of these biomarkers with IBD pathogenesis. The composition and function of bacteria and fungi in stool from 100 IBD patients and 97 controls were profiled using next-generation sequencing. We evaluated the cumulative risk of relapse according to bacterial and fungal enterotypes. The microbiome and mycobiome alpha diversity in IBD patients were significantly lower and higher than in the controls, respectively; the micro/mycobiome beta diversity differed significantly between IBD patients and the controls. *Ruminococcus gnavus*, *Cyberlindnera jadinii*, and *Candida tropicalis* increased in IBD patients. Combining functional and species analyses revealed that lower sugar import and higher modified polysaccharide production were associated with IBD pathogenesis. Tricarboxylic acid cycling consuming acetyl CoA was higher in IBD patients than the controls, leading to lower short-chain fatty acid (SCFA) fermentation. Bacterial and fungal enterotypes were not associated with IBD relapse. We found differences in bacterial and fungal species between IBD patients and controls. A working model for the role of gut bacteria in IBD pathogenesis is proposed, wherein bacterial species increase modified N-glycan production and decrease SCFA fermentation.

## 1. Introduction

The pathophysiology of inflammatory bowel disease (IBD) is complex. Although most studies agree that the gut microbiota plays a crucial role, further research is required. With advances in next-generation sequencing, numerous studies have recently been conducted on gut bacteria. However, most studies simply compare gut microbiomes between patients and healthy controls. This leads to varying results and conflicting inferences [1]. Therefore, further research is needed to analyze the functional changes and metabolites of gut bacteria, which may be more crucial than their simple composition [2]. Although microbial biomarkers that can distinguish IBD patients from healthy individuals are essential to elucidate the pathogenesis of IBD, specific gut bacteria or enterotypes that can predict prognosis are more useful for clinicians and patient treatment. There are few studies on this topic, most are retrospective, not adding any value to clinical treatment or patient care [3,4].

To date, gut microbiome analyses have mainly focused on bacteria. However, as the role of intestinal fungi has recently been highlighted, the mycobiome is also being emphasized for IBD [5]. In IBD patients, the diversity of intestinal fungi increases compared to that of intestinal bacteria, and so does the presence of *Candida albicans* [6,7]. However, fungi account for a small proportion of intestinal microbes compared to gut bacteria, and sequencing mycobiomes is challenging. Therefore, studies on the mycobiome in IBD patients are scarce, and the results vary [8]. In addition, most previous studies were conducted with <30 patients, overwhelmingly Westerners, which is a very narrow sample space. Therefore, these studies provide limited value for IBD treatment. In addition, there are few studies on fungal enterotypes and the effect of mycobiomes on IBD prognosis.

To overcome some of these limitations, we prospectively performed bacterial and fungal metagenomic studies for IBD patients and tried to address the functional implications of the biomarkers and predictability of IBD relapse.

## 2. Materials and Methods

### 2.1. Patients

Stool samples and clinical data were prospectively collected from 100 IBD patients enrolled in the Seoul National University Bundang Hospital IBD cohort between March 2018 and July 2020. Patients with IBD-unclassified were excluded. Each patient received the standard treatment for IBD [9,10]. Active disease was defined as a fecal calprotectin ≥250 mg/kg on the day of stool collection. Relapse was defined as a composite outcome of (i) new use of steroids, immunomodulators, and biologics; (ii) a visit to an emergency department; and (iii) hospitalization. Stool samples for the age- and sex-matched control group were acquired from the Korean gut microbiome bank study (B-1701-380-304); this study aimed to evaluate gut microbiome of healthy Korean individuals. Therefore, individuals with acute or chronic illness, a history of cancer within 5 years, and a history of antibiotic or probiotic use within 3 months were excluded. Because they were unable to find age-matched controls for three old patients with ulcerative colitis (UC), a total of 97 control samples were collected. This study was approved by the Institutional Review Board of Seoul National University Bundang Hospital (IRB No.:B-2106-693-304). All stool samples were frozen in a −70 °C freezer immediately after collection.

### 2.2. DNA Extraction and Sequencing for Microbiome and Mycobiome Analysis

For microbiome and mycobiome analysis, total DNA was extracted from stool samples using a Maxwell RSC PureFood GMO and Authentication Kit (Promega, Seoul, Republic of Korea). For microbiome analysis, PCR amplification was performed for the V3–V4 regions of the 16S rRNA gene. Sequencing was performed using the MiSeq Sequencing System (Illumina; San Diego, CA, USA). Primary microbiome analyses were conducted using previously described procedures [11,12,13]. Briefly, processing raw reads started with a quality check and filtering of low-quality reads by Trimmomatic version 0.32. Paired-end sequence data were merged using VSEARCH version 2.13.4. Primers were trimmed with the alignment algorithm of Myers and Miller at a similarity cut-off of 0.8. Non-specific amplicons were detected in HMMER software package version 3.2.1. Unique reads were extracted, and redundant reads clustered with the unique reads. The EzBioCloud 16S rRNA database was used for taxonomic assignment, followed by more precise pairwise alignment. Chimeric reads were filtered using the UCHIME algorithm and the non-chimeric 16S rRNA database. Reads that were not identified to the species level (with <97% similarity) were compiled, and the cluster fast command was used to perform de novo clustering to generate additional operational taxonomic units (OTUs). Finally, OTUs with single reads (singletons) were omitted from further analysis. The OTU abundance was normalized to a read count of 10,000.

For mycobiome analysis, polymerase chain reaction (PCR) amplification was performed using fusion primers targeting the internal transcribed spacer (ITS) 2 region with the extracted DNA. Sequencing was performed using an Illumina MiSeq Sequencing System. Read pairs on the overlapping regions of sequences were joined using VSEARCH version 2.13.4 [14]. Reads were quality-checked and filtered by removing low-quality reads with the FASTX-Toolkit. Unique reads were extracted, and redundant reads were clustered with the unique reads using VSEARCH version 2.13.4. Short reads (<100 bp) and singletons were removed before clustering at a user-defined threshold (97% sequence identity) using VSEARCH. The resulting representative sequences for each cluster were subjected to chimera detection and filtered using the UNITE UCHIME reference dataset (UNITE_General_s_01.12.2017) [15]. The input ITS sequences were mapped onto the chimera-free representative sequences at the defined threshold. These representatives were taxonomically assigned with the RDP Classifier against the UNITE fungal ITS reference dataset [16,17]. The OTU abundance was normalized to a read count of 5000.

### 2.3. Statistical Analyses

Diversity calculation and biomarker discovery were conducted using in-house programs (CJ Bioscience, Inc., Seoul, Republic of Korea). For alpha diversity analysis, the ACE [18], Shannon [19], Simpson [19], and phylogenetic diversity [20] indices were calculated. For beta diversity analysis, the overall phylogenetic distance between communities was estimated and visualized using Jensen–Shannon-based principal coordinate analysis [21]. Permutational multivariate analysis of variance (PERMANOVA) was used to evaluate differences between groups. Linear discriminant analysis effect size (LEfSe) analysis was conducted to determine the features that most likely explained the differences between groups by coupling standard tests for statistical significance with additional tests encoding biological consistency and effect relevance [22]. To predict the functional profiling of the microbiome, phylogenetic community investigation was performed via unobserved state reconstruction (PICRUSt) using Kyoto Encyclopedia of Genes and Genomes (KEGG) orthology [23]. Cluster analysis based on bacterial and fungal species compositions was performed to classify the subjects into enterotypes [24]. All microbiome and mycobiome analyses were performed using non-parametric methods, and the results were considered statistically significant at *p*-values < 0.05. False discovery rate (FDR)-adjusted *p*-values were applied to the LEfSe analyses. All analytics mentioned above were performed in EzBioCloud (www.ezbiocloud.net (accessed on 7 July 2022)).

We compared the cumulative relapse risk according to bacterial and fungal enterotypes using Kaplan–Meier survival analysis and the log-rank test. Cox proportional hazards regression was performed to evaluate independent predictors of relapse in IBD patients. Variables with *p*-value < 0.2 in univariable analysis were included in the multivariable Cox proportional hazards regression model. All analyses were performed using Stata version 16.0 (StataCorp LLC; College Station, TX, USA). Statistical significance was set at *p* < 0.05.

## 3. Results

### 3.1. Comparison of Stool Microbiomes between IBD Patients and Healthy Controls

Sixty-seven patients with UC and 33 patients with Crohn’s disease were enrolled in this study. The characteristics of IBD patients and the control group are shown in Table S1. The median age of IBD patients and the controls was 38 and 36 years old, respectively. Males constituted 77.0% and 76.3% of IBD patients and controls, respectively.

**Figure 1:**
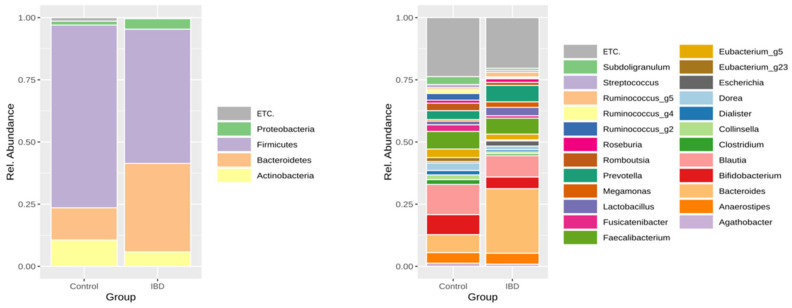
Taxonomic composition of the stool microbiome of patients with inflammatory bowel disease and healthy controls (left: phylum level, right: genus level).

The taxonomic composition of the stool microbiome of patients with IBD and controls is shown in Figure 1. Based on LEfSe analysis, the abundance of *Bacteroidetes* (35.56% vs. 12.92%, *p* = 1.86 × 10^−8^), *Proteobacteria* (4.22% vs. 1.57%, *p* = 0.00685), and *Fusobacteria* (0.004% vs.<0.001%, *p* = 0.00039) significantly increased, while that of *Firmicutes* (53.95% vs. 73.46%, *p* = 6.00 × 10^−8^), *Actinobacteria* (5.83% vs. 10.62%, *p* = 0.00023), and *Verrucomicrobia* (<0.001% vs. 1.15%, *p* = 1.86 × 10^−8^) significantly decreased in IBD patients compared to the controls.

We identified 48 species as biomarkers for differentiating IBD from controls (Table S2). Compared to the control group, the abundance of 10 species increased in IBD patients while that of 38 species decreased. The prevalence of pathobionts such as *Escherichia coli* and *Ruminococcus gnavus* increased, while that of beneficial bacteria such as *Akkermansia muciniphila* and the *Bifidobacterium* groups decreased in patients with IBD.

**Figure 2:**
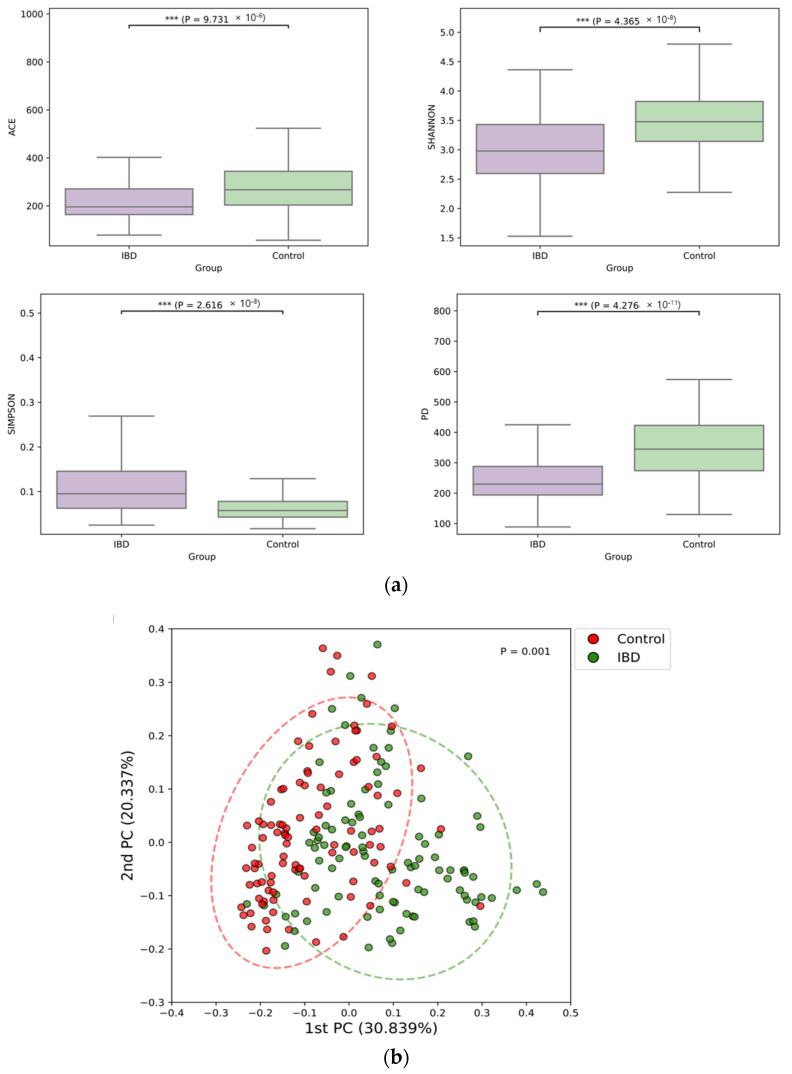
Alpha-diversity (**a**) and beta-diversity (**b**) of the stool microbiomes of patients with inflammatory bowel disease and healthy controls.

The microbiome alpha diversity in IBD patients was significantly lower than that in the controls (Figure 2a). Moreover, beta diversity significantly differed between the two groups (Figure 2b).

### 3.2. Functional Analysis of the Stool Microbiome between IBD Patients and Healthy Controls

Having explored the species characteristics of IBD, we investigated the functional aspects of the species using PICRUSt analysis. Three and one functional pathways were predicted to significantly increase and decrease in patients with IBD compared to the controls, respectively (Table 1).

In the KEGG pathways, “KO01100 metabolic pathways” indicates that there were broad metabolic differences. Specifically, the 00520 “amino sugar and nucleotide sugar metabolism” pathway involves the synthesis of uracil-diphosphate-glucose and N-acetyl muraminic acid from monosaccharides, such as glucose. This pathway generates amino or nucleotide sugars used for membrane glycosylation or polymeric glycosides, such as peptidoglycans, in the bacterial membrane [25,26,27]. An increase in this metabolism may lead to higher monosaccharide consumption for polymeric glycosides synthesis, and this process may increase glycosylated membrane components [28]. The “00511 other glycan degradation” pathway involves N-glycan and glycolipid biosynthesis in the KEGG pathway annotation. This metabolic pathway is consistent with the above “00520 amino sugar and nucleotide sugar metabolism” pathway, in that both work together to generate glycosylated polymers, typically found in the cell wall components of bacteria such as peptidoglycan and lipid A [27,28]. Additionally, it may be involved in the synthesis of glycoproteins secreted from cells. Notably, this suggestion is supported by the “M00060 lipopolysaccharide biosynthesis, KDO2-lipid A” module (Table S3). For “02010 ABC transporters”, to interpret the metabolic steps is challenging, since they involve many different transporters, including sugar molecule importers [29]. Nevertheless, decreased “M00196 raffinose/stachyose/melibiose transport system” module activity correlates with the decrease in the number of sugar molecules in the bacterial cells.

Other PICRUSt (module) analyses also showed interesting metabolic behaviors, such as increased “M00011 citrate cycle, second carbon oxidation, 2-oxoglutarate à oxaloacetate” and “M00620 incomplete reductive citrate cycle, acetyl coenzyme A (acetyl-CoA) à oxoglutarate.” These two modules essentially refer to the two irreversible steps of the tricarboxylic acid (TCA) cycle [30]. An increase in these steps would enhance the overall activity of the TCA cycle. Combining the two modules yields an increase in the overall consumption of acetyl-CoA through acetyl-CoA à oxoglutarate à oxaloacetate metabolism [30,31]. This has a crucial effect on short-chain fatty acid (SCFA) fermentation as butyrate is produced from two acetyl-CoA molecules [32]. Therefore, acetyl-CoA consumption through the TCA cycle should decrease SCFA generation, leading to lower SCFA levels [33,34,35]. This suggestion is supported by an increase in the “M0144 NADH: quinone oxidoreductase, prokaryotes” module, because the enhanced TCA cycle generates NADH, which should be reduced by NADH oxidase rather than by SCFA fermentation. The overall metabolic changes and specific KEGG modules relevant in IBD are described in Figure 5 (see Figure 5 below).

### 3.3. Comparison of the Stool Mycobiome between IBD Patients and Healthy Controls

**Figure 3:**
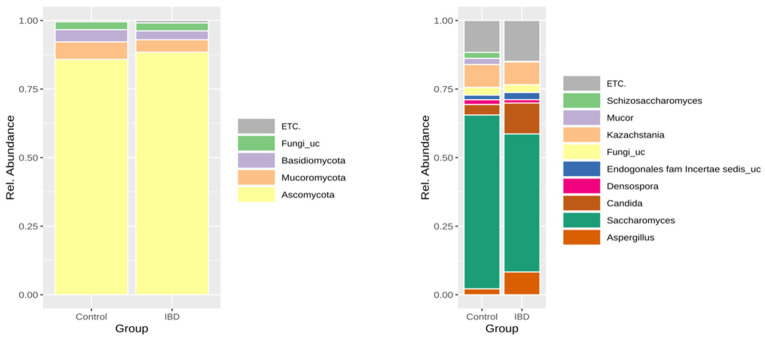
Taxonomic composition of the stool mycobiome of patients with inflammatory bowel disease and healthy controls (left: phylum level, right: genus level).

The sequencing yield of the mycobiome was lower than that of the microbiome; successful sequencing was possible in 58% and 41% of patients with IBD and the control group, respectively. The taxonomic composition of the stool mycobiome of patients with IBD and the control group is shown in Figure 3.

There was no phylum that showed significantly different abundance between IBD patients and the control group. Based on LEfSe analysis, the abundance of *Cyberlindnera jadinii*, *Candida tropicalis*, and *Saccharomycetes* sp. strain KP196597 significantly increased in IBD patients compared to the control group (Table 2).

**Figure 4:**
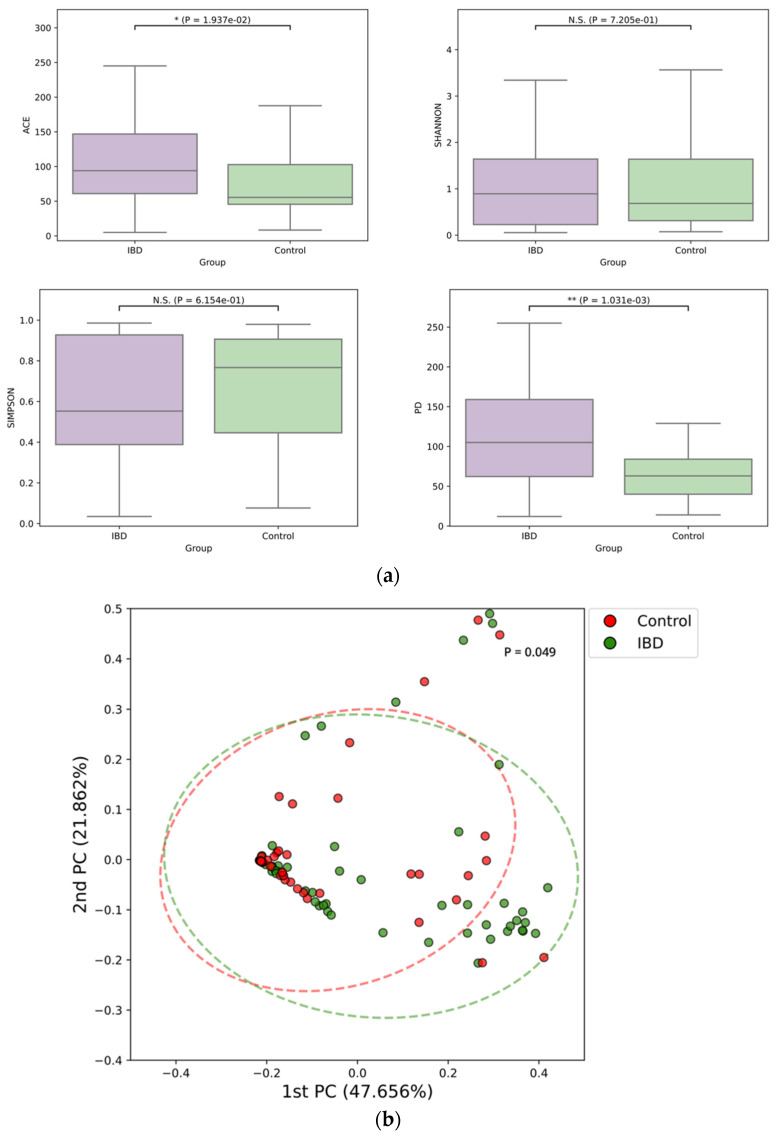
Alpha-diversity (**a**) and beta-diversity (**b**) of the stool mycobiome of patients with inflammatory bowel disease and healthy controls.

Among the alpha diversity indices, the ACE index and phylogenetic diversity of the mycobiome were significantly higher in patients with IBD than in the control group (Figure 4a). Moreover, beta diversity significantly differed between the groups (Figure 4b).

### 3.4. Comparison of the Stool Microbiome and Mycobiome According to the Disease Activity of IBD

IBD patients were divided into two groups: active disease (N = 49) and inactive disease (N = 51). When we compared the stool microbiome (Figure S1) and mycobiome (Figure S2) of these two groups, there were no differences in the alpha or beta diversities. In addition, no microbiome or mycobiome was differentially expressed according to the disease activity on LEfSe analysis.

### 3.5. Enterotypes Based on the Stool Microbiome and Mycobiome, and Relapse Risk Factors for IBD Patients

Using cluster analysis, IBD patients were divided into two enterotypes based on their stool microbiome (enterotype 1: N = 80, enterotype 2: N = 20) (Figure S3a). Clinical characteristics such as age, sex, smoking status, disease duration, fecal calprotectin, and exposed medications did not differ between bacterial enterotypes (data available on request). The IBD patients were followed up for a median of 33 months. There was no significant difference in relapse risk between enterotypes 1 and 2 based on the stool microbiome (Figure S3b). In multivariable analysis, an elevated fecal calprotectin (≥250 mg/kg) was the only significant risk factor associated with relapse (Table S4).

In the cluster analysis of the stool mycobiome, the Calinski–Harabasz index was the highest and the second highest when IBD patients were divided into 20 and 19 clusters, respectively (Figure S4a). However, dividing 100 patients into 19–20 enterotypes is not clinically practical. Therefore, we selected the third highest Calinski–Harabasz index, dividing the IBD patients into three enterotypes (Figure S4b). There was no significant difference in relapse risk between enterotypes (log-rank test, *p* = 0.435) (Figure S4c). In the multivariate analysis, there were no significant risk factors associated with relapse (data available on request).

## 4. Discussion

We identified microbial, mycotic, and functional biomarkers to differentiate IBD patients from healthy controls. Combining functional and species analyses, we suggest two metabolic features for the role of gut bacteria in IBD pathogenesis. However, bacterial and fungal enterotypes cannot be used to predict relapse in IBD patients. In future studies, deep analyses of the stool microbiome and mycobiome, up to the strain level, using shotgun sequencing are needed to elucidate the true value of bacterial and fungal enterotypes in the prediction of relapse in IBD patients.

The decrease in beneficial bacteria, such as *A. muciniphila* and the *Bifidobacterium* groups, along with the increase in pathobionts, such as *E. coli* and *R. gnavus*, observed in this study are consistent with previous studies [36,37,38,39]. In addition, the decrease in the alpha diversity of the microbiome in IBD patients and the difference in the beta diversity between them and the controls are also well known [40]. Notably, species such as *Collinsella*, *Prevotella*, and *Gemmiger*, belonging to genera abundant in rural Chinese people, decreased in IBD patients, while *E. coli*, which is abundant in urban people, increased [41]. In addition, the prevalence of *Proteus mirabilis* increased in IBD patients. *Proteus mirabilis* plays a crucial role in the pathogenesis of IBD [42] and is increased in people who consume substantial amounts of polysorbate-80, an emulsifier commonly used to make processed foods [43]. Furthermore, the abundance of *Bifidobacterium* groups that have an anti-inflammatory role in the human gut [44] decreases in IBD patients, while that of *R. gnavus*, which has a pro-inflammatory role, increases [45]. These two bacterial species are known to be abundant in unprocessed and ultra-processed foods, respectively. Taken together, these results suggest that the change in gut microbiota following urbanization and increased consumption of processed foods in Asian countries may be related to the rising incidences of IBD [46].

**Figure 5:**
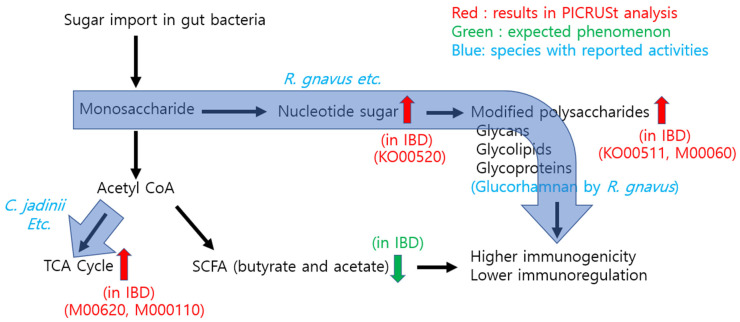
Working model for the role of the microbiome in the pathogenesis of inflammatory bowel disease through which bacteria modulate host immunity.

Notably, the consistent themes of the PICRUSt analyses suggest an increase in modified polysaccharides and a decrease in SCFAs. In terms of IBD pathogenesis, these metabolic features can be interpreted in two ways: first, an increase in modified polysaccharides, particularly lipid A or cell wall peptidoglycan, can contribute to higher bacterial immunogenicity in the IBD group. Second, a decrease in unmodified sugar molecules and SCFAs could lead to different host immune responses and enterocyte energy metabolism [47]. For the analysis of bacterial species, we observed an increase in the abundance of *R. gnavus* in IBD patients. Notably, a recent study showed that these bacteria secrete glucorhamnan polysaccharide, which induces TNF-α secretion from host dendritic cells, thereby eliciting an immune response [48]. As glucorhamnan polysaccharide is a modified polysaccharide, this microbial analysis is consistent with our PICRUSt results suggesting higher levels of modified polysaccharides in IBD patients. As this previous study was performed in vitro, our results support this finding through the functional analysis of metagenomic data from IBD patients. Combining these functional and species analyses, we suggest a working model through which bacteria modulate host immunity during IBD pathogenesis (Figure 5).

In the gut of IBD patients, bacteria have low sugar import and high modified polysaccharide levels that can directly generate higher immunogenicity. In addition, an increased TCA cycle consumes more acetyl-CoA, leading to lower SCFA fermentation and subsequent host immune modulation. It should be noted that our discussion on SCFA is based on our PICURSt analysis, and it warrants further experimental validation, i.e., measurements of SCFA from fecal samples, for confirmation.

An increase in *C. tropicalis* in IBD patients has been previously reported [49,50]. However, the fact that *C. jadinii*, previously known as *Candida utilis*, exhibited the largest increase in abundance in IBD patients is interesting. One study also suggested an increase in this fungus in pediatric patients with IBD [51]. *Cyberlindnera jadinii* has been widely used as a flavoring additive in processed foods. Excessive consumption of processed foods is closely associated with low consumption of microbiota-accessible carbohydrates [52], and these might lead to the development of IBD. Whether the increase in *C. jadinii* in IBD patients is related to an increased intake of processed foods or whether actual colonization affects host immunity is unclear. *Cyberlindnera jadinii* is negative for the crab-tree effect and has one of the highest respiratory capacities among the characterized yeast species [53]. Therefore, this fungus prefers respiration to fermentation to generate energy. Blooms of this fungus may also contribute to a decrease in the fermentation of indigestible fiber and a consequent decrease in SCFA production in IBD patients.

However, whether the gut microbiome differs depending on the degree of disease activity remains controversial [54,55]. In this study, there was no difference in the alpha and beta diversity of the microbiome and mycobiome according to the disease activity of IBD patients, despite evaluating disease activity using fecal calprotectin, which is highly sensitive in detecting inflammation in the gut. Inversely, since there was no correlation between disease activity and the micro/mycobiome, we expected it to be very useful if enterotypes could independently predict prognosis in IBD patients. However, bacterial and fungal enterotypes were not significantly associated with clinical relapse in IBD patients. Several studies have investigated whether the gut microbiome can predict clinical relapse in IBD; however, the findings are varied [56,57]. Very few studies have attempted to predict clinical relapse in IBD patients using microbial or fungal enterotypes. Regarding the gut mycobiome, it is difficult to define the enterotypes themselves. The reason for this issue is that the composition of fungi is more varied and unstable than that of bacteria [58]. In addition, many factors affect the clinical outcomes and relapse of IBD. Taken together, at present, clinical factors such as fecal calprotectin levels are still more powerful than complex microbiomes and mycobiomes in predicting clinical relapse.

This study has several strengths. First, because the results of functional and composition analyses in the microbiome were consistent and agreed with each other, we could generate a working model for the role of microbes in IBD pathogenesis. The results of fungal analysis further supported our hypothesis. Second, to our knowledge, this is the first study to predict the prognosis of patients with IBD using fungal enterotypes. In addition, important clinical factors that affect the course of IBD were prospectively collected from a well-established cohort. The sample size was larger than that in previous studies, and the patients were followed up for years.

However, this study has limitations. First, PICRUSt only predicts metagenome functional content. Therefore, the changes in the metabolic pathways in this study need to be further validated. Second, functional analysis of the mycobiome could not be performed because the bioinformatics platform used did not support this analysis. In addition, the yield of fungal DNA extraction for mycobiome analysis was low both in IBD patients and the controls, which may have led to a lower verification power for survival analysis. The low fungal yield in stool is not a problem in our experimental technique but is an inherent difficulty in fungal analysis. To proceed with next-generation sequencing, a certain amount of DNA must be secured in samples. However, the number of fungi present in the stool is small compared to that of bacteria [59]. In addition, the cell wall of fungi is harder than that of bacteria, so it is not easily broken. Third, the heterogeneity of the patients was large compared to the number of patients. Therefore, we could not evaluate the microbiome and mycobiome according to specific disease phenotypes, such as disease behavior in Crohn’s disease (CD) and disease extent in UC. When IBD patients were divided into the UC and CD groups and compared with the controls, we found that the bacterial composition of UC was between that of CD and the control in the principal coordinate analysis plot. However, the difference was not statistically significant (data available on request). Fourth, the information about diet, lifestyle, and genetic risk factors for IBD was not collected in IBD patients and the controls. Data about the use of antibiotics in IBD patients is missing. All these factors can affect gut microbiota and are associated with the development of IBD. Finally, we used stool samples in this study. The stool is a mixture of the distal gut microbiota and is not fully representative of the gut microbiota specific to a particular region. Therefore, fecal microbiota may differ from mucosa-associated microbiota acquired from tissue samples. This might have contributed to the absence of *Malassezia* species, reported to be associated with CD [60].

## 5. Conclusions

In conclusion, bacterial and fungal dysbiosis was observed in the stool of IBD patients. We identified microbial, mycotic, and functional biomarkers for differentiating IBD patients from healthy controls and suggested a working model for the metabolic role of gut bacteria in IBD pathogenesis. In contrast, the fecal microbiome and mycobiome were not found to be associated with IBD relapse.

## Figures and Tables

**Table 1:** Functional pathways showing a significant difference in expression in the stool microbiome between patients with inflammatory bowel disease and healthy controls.

KEGG Pathway	Definition	LDA Effect Size *	FDR Adjusted *p*-Value
Increased in IBD compared to the control			
KO01100	Metabolic pathways	3.490	<0.001
KO00511	Other glycan degradation	3.062	<0.001
KO00520	Amino sugar and nucleotide sugar metabolism	3.047	<0.001
Decreased in IBD compared to the control			
KO02010	ABC transporters	3.194	<0.001

FDR, false discovery rate; IBD, inflammatory bowel disease; KEGG, Kyoto Encyclopedia of Genes and Genomes; LDA, linear discriminant analysis. * Only taxa showing LDA effect size ≥3.0 are presented.

**Table 2:** Taxa showing a significant difference in abundance in the stool mycobiome between patients with inflammatory bowel disease and healthy control (species level).

Taxon Name	LDA Effect Size	FDR Adjusted *p*-Value
Species more abundant in IBD than in controls		
*Cyberlindnera jadinii*	3.305	0.006
*Candida tropicalis*	2.832	0.049
*Saccharomycetes* sp_KP196597	2.718	0.028

FDR, false discovery rate; IBD, inflammatory bowel disease; LDA, linear discriminant analysis.

## Data Availability

Sequencing data of the microbiome/mycobiome in patients with IBD and the mycobiome in the control group were deposited in the sequence read archive of the National Center for Biotechnology Information, under accession number PRJNA968150. Because the sequencing data of the microbiome in the control group is a part of the Korean gut microbiome bank study and the main results of this study have not been published yet, we cannot deposit this data in a public database; this data is available from H.Y. upon request.

## Supplementary Materials

The following supporting information can be downloaded at: https://www.mdpi.com/article/10.3390/microorganisms11122882/s1, Figure S1: Alpha-diversity (a) and beta-diversity (b) of the stool microbiome of patients with inflammatory bowel disease, according to disease activity; Figure S2: Alpha-diversity (a) and beta-diversity (b) of the stool mycobiome of patients with inflammatory bowel disease, according to disease activity; Figure S3: (a) Enterotype of patients with inflammatory bowel disease based on the stool microbiome, (b) Comparison of Kaplan–Meier survival curves according to bacterial enterotypes; Figure S4: (a) Calinski Harabasz indices for cluster analysis of stool fungal enterotypes, (b) enterotype of patients with inflammatory bowel disease based on the stool mycobiome, and (c) comparison of Kaplan–Meier survival curves according to fungal enterotypes; Table S1: Baseline characteristics of patients with inflammatory bowel disease and the control group; Table S2: Taxa showing a significant difference in the abundance in the stool microbiome between patients with inflammatory bowel disease and healthy control (species level); Table S3: Functional modules showing a significant difference in expression in the stool microbiome between patients with inflammatory bowel disease and healthy controls; Table S4: Univariable and multivariable analyses of predictors associated with relapse in patients with inflammatory bowel disease

## References

[1] Aldars-García L., Chaparro M., Gisbert J.P. (2021). Systematic review: The gut microbiome and its potential clinical application in inflammatory bowel disease. Microorganisms.

[2] Morgan X.C., Tickle T.L., Sokol H., Gevers D., Devaney K.L., Ward D.V., Reyes J.A., Shah S.A., LeLeiko N., Snapper S.B. (2012). Dysfunction of the intestinal microbiome in inflammatory bowel disease and treatment. Genome Biol..

[3] Shin S.Y., Kim Y., Kim W.S., Moon J.M., Lee K.M., Jung S.A., Park H., Huh E.Y., Kim B.C., Lee S.C. (2023). Compositional changes in fecal microbiota associated with clinical phenotypes and prognosis in Korean patients with inflammatory bowel disease. Intest. Res..

[4] Park S.K., Kim H.N., Choi C.H., Im J.P., Cha J.M., Eun C.S., Kim T.O., Kang S.B., Bang K.B., Kim H.G. (2020). Differentially abundant bacterial taxa associated with prognostic variables of Crohn’s disease: Results from the Impact study. J. Clin. Med..

[5] Iliev I.D. (2022). Mycobiota-host immune interactions in IBD: Coming out of the shadows. Nat. Rev. Gastroenterol. Hepatol..

[6] Sokol H., Leducq V., Aschard H., Pham H.P., Jegou S., Landman C., Cohen D., Liguori G., Bourrier A., Nion-Larmurier I. (2017). Fungal microbiota dysbiosis in IBD. Gut.

[7] Imai T., Inoue R., Kawada Y., Morita Y., Inatomi O., Nishida A., Bamba S., Kawahara M., Andoh A. (2019). Characterization of fungal dysbiosis in Japanese patients with inflammatory bowel disease. J. Gastroenterol..

[8] Guzzo G.L., Andrews J.M., Weyrich L.S. (2022). The neglected gut microbiome: Fungi, Protozoa, and bacteriophages in inflammatory bowel disease. Inflamm. Bowel Dis..

[9] Koh S.J., Hong S.N., Park S.K., Ye B.D., Kim K.O., Shin J.E., Yoon Y.S., Lee H.S., Jung S.H., Choi M. (2023). Korean clinical practice guidelines on biologics for moderate to severe Crohn’s disease. Intest. Res..

[10] Na S.Y., Choi C.H., Song E.M., Bang K.B., Park S.H., Kim E.S., Park J.J., Keum B., Lee C.K., Lee B.I. (2023). Korean clinical practice guidelines on biologics and small molecules for moderate-to-severe ulcerative colitis. Intest. Res..

[11] Chun J., Kim K.Y., Lee J.H., Choi Y. (2010). The analysis of oral microbial communities of wild-type and toll-like receptor 2-deficient mice using a 454 GS FLX Titanium pyrosequencer. BMC Microbiol..

[12] Hur M., Kim Y., Song H.R., Kim J.M., Choi Y.I., Yi H. (2011). Effect of genetically modified poplars on soil microbial communities during the phytoremediation of waste mine tailings. Appl. Environ. Microbiol..

[13] Kim B.S., Kim J.N., Yoon S.H., Chun J., Cerniglia C.E. (2012). Impact of enrofloxacin on the human intestinal microbiota revealed by comparative molecular analysis. Anaerobe.

[14] Rognes T., Flouri T., Nichols B., Quince C., Mahé F. (2016). VSEARCH: A versatile open source tool for metagenomics. PeerJ.

[15] Edgar R.C., Haas B.J., Clemente J.C., Quince C., Knight R. (2011). UCHIME improves sensitivity and speed of chimera detection. Bioinformatics.

[16] Wang Q., Garrity G.M., Tiedje J.M., Cole J.R. (2007). Naive Bayesian classifier for rapid assignment of rRNA sequences into the new bacterial taxonomy. Appl. Environ. Microbiol..

[17] Nilsson R.H., Larsson K.H., Taylor A.F.S., Bengtsson-Palme J., Jeppesen T.S., Schigel D., Kennedy P., Picard K., Glöckner F.O., Tedersoo L. (2019). The UNITE database for molecular identification of fungi: Handling dark taxa and parallel taxonomic classifications. Nucleic Acids Res..

[18] Chao A., Lee S.-M. (1992). Estimating the number of classes via sample coverage. J. Am. Stat. Assoc..

[19] Magurran A.E. (2013). Measuring Biological Diversity.

[20] Faith D.P. (1992). Conservation evaluation and phylogenetic diversity. Biol. Conserv.

[21] Lin J. (1991). Divergence measures based on the Shannon entropy. IEEE Trans. Inf. Theory.

[22] Segata N., Izard J., Waldron L., Gevers D., Miropolsky L., Garrett W.S., Huttenhower C. (2011). Metagenomic biomarker discovery and explanation. Genome Biol..

[23] Langille M.G., Zaneveld J., Caporaso J.G., McDonald D., Knights D., Reyes J.A., Clemente J.C., Burkepile D.E., Vega Thurber R.L., Knight R. (2013). Predictive functional profiling of microbial communities using 16S rRNA marker gene sequences. Nat. Biotechnol..

[24] Arumugam M., Raes J., Pelletier E., Le Paslier D., Yamada T., Mende D.R., Fernandes G.R., Tap J., Bruls T., Batto J.M. (2011). Enterotypes of the human gut microbiome. Nature.

[25] De Bruyn F., Maertens J., Beauprez J., Soetaert W., De Mey M. (2015). Biotechnological advances in UDP-sugar based glycosylation of small molecules. Biotechnol. Adv..

[26] Nothaft H., Szymanski C.M. (2010). Protein glycosylation in bacteria: Sweeter than ever. Nat. Rev. Microbiol..

[27] Egan A.J.F., Errington J., Vollmer W. (2020). Regulation of peptidoglycan synthesis and remodelling. Nat. Rev. Microbiol..

[28] Whitfield C., Trent M.S. (2014). Biosynthesis and export of bacterial lipopolysaccharides. Annu. Rev. Biochem..

[29] Thomas C., Tampé R. (2020). Structural and mechanistic principles of ABC transporters. Annu. Rev. Biochem..

[30] Mailloux R.J., Bériault R., Lemire J., Singh R., Chénier D.R., Hamel R.D., Appanna V.D. (2007). The tricarboxylic acid cycle, an ancient metabolic network with a novel twist. PLoS ONE.

[31] Malloy C.R., Sherry A.D., Jeffrey F.M. (1990). Analysis of tricarboxylic acid cycle of the heart using 13C isotope isomers. Am. J. Physiol..

[32] Duncan S.H., Barcenilla A., Stewart C.S., Pryde S.E., Flint H.J. (2002). Acetate utilization and butyryl coenzyme A (CoA):acetate-CoA transferase in butyrate-producing bacteria from the human large intestine. Appl. Environ. Microbiol..

[33] Molenaar D., van Berlo R., de Ridder D., Teusink B. (2009). Shifts in growth strategies reflect tradeoffs in cellular economics. Mol. Syst. Biol..

[34] Pfeiffer T., Schuster S., Bonhoeffer S. (2001). Cooperation and competition in the evolution of ATP-producing pathways. Science.

[35] Thauer R.K., Jungermann K., Decker K. (1977). Energy conservation in chemotrophic anaerobic bacteria. Bacteriol. Rev..

[36] Derrien M., Belzer C., de Vos W.M. (2017). *Akkermansia muciniphila* and its role in regulating host functions. Microb. Pathog..

[37] Prosberg M., Bendtsen F., Vind I., Petersen A.M., Gluud L.L. (2016). The association between the gut microbiota and the inflammatory bowel disease activity: A systematic review and meta-analysis. Scand. J. Gastroenterol..

[38] Zhao Y., Chen L., Chen L., Huang J., Chen S., Yu Z. (2022). Exploration of the potential relationship between gut microbiota remodeling under the influence of high-protein diet and Crohn’s disease. Front. Microbiol..

[39] Hu S., Png E., Gowans M., Ong D.E.H., de Sessions P.F., Song J., Nagarajan N. (2021). Ectopic gut colonization: A metagenomic study of the oral and gut microbiome in Crohn’s disease. Gut Pathog..

[40] Matsuoka K., Kanai T. (2015). The gut microbiota and inflammatory bowel disease. Semin. Immunopathol..

[41] Sun S., Wang H., Howard A.G., Zhang J., Su C., Wang Z., Du S., Fodor A.A., Gordon-Larsen P., Zhang B. (2022). Loss of novel diversity in human gut microbiota associated with ongoing urbanization in China. mSystems.

[42] Zhang J., Hoedt E.C., Liu Q., Berendsen E., Teh J.J., Hamilton A., O’ Brien A.W., Ching J.Y.L., Wei H., Yang K. (2021). Elucidation of *Proteus mirabilis* as a key Bacterium in Crohn’s disease inflammation. Gastroenterology.

[43] Srour B., Kordahi M.C., Bonazzi E., Deschasaux-Tanguy M., Touvier M., Chassaing B. (2022). Ultra-processed foods and human health: From epidemiological evidence to mechanistic insights. Lancet Gastroenterol. Hepatol..

[44] Wu H., Chen X., Zhang S., Li J. (2022). Gut microbiota, the potential biological medicine for prevention, intervention and drug sensitization to fight diseases. Nutrients.

[45] Bolte L.A., Vich Vila A., Imhann F., Collij V., Gacesa R., Peters V., Wijmenga C., Kurilshikov A., Campmans-Kuijpers M.J.E., Fu J. (2021). Long-term dietary patterns are associated with pro-inflammatory and anti-inflammatory features of the gut microbiome. Gut.

[46] Shim J.S., Shim S.Y., Cha H.J., Kim J., Kim H.C. (2021). Socioeconomic characteristics and trends in the consumption of ultra-processed foods in Korea from 2010 to 2018. Nutrients.

[47] Kostic A.D., Xavier R.J., Gevers D. (2014). The microbiome in inflammatory bowel disease: Current status and the future ahead. Gastroenterology.

[48] Henke M.T., Kenny D.J., Cassilly C.D., Vlamakis H., Xavier R.J., Clardy J. (2019). *Ruminococcus gnavus*, a member of the human gut microbiome associated with Crohn’s disease, produces an inflammatory polysaccharide. Proc. Natl. Acad. Sci. USA.

[49] Hoarau G., Mukherjee P.K., Gower-Rousseau C., Hager C., Chandra J., Retuerto M.A., Neut C., Vermeire S., Clemente J., Colombel J.F. (2016). Bacteriome and mycobiome interactions underscore microbial dysbiosis in familial Crohn’s disease. mBio.

[50] Krawczyk A., Salamon D., Kowalska-Duplaga K., Bogiel T., Gosiewski T. (2021). Association of fungi and Archaea of the gut microbiota with Crohn’s disease in pediatric patients-pilot study. Pathogens.

[51] Chehoud C., Albenberg L.G., Judge C., Hoffmann C., Grunberg S., Bittinger K., Baldassano R.N., Lewis J.D., Bushman F.D., Wu G.D. (2015). Fungal signature in the gut microbiota of pediatric patients with inflammatory bowel disease. Inflamm. Bowel Dis..

[52] Sonnenburg J.L., Sonnenburg E.D. (2019). Vulnerability of the industrialized microbiota. Science.

[53] Sousa-Silva M., Vieira D., Soares P., Casal M., Soares-Silva I. (2021). Expanding the knowledge on the skillful yeast *Cyberlindnera jadinii*. J. Fungi..

[54] Galazzo G., Tedjo D.I., Wintjens D.S.J., Savelkoul P.H.M., Masclee A.A.M., Bodelier A.G.L., Pierik M.J., Jonkers D.M.A.E., Penders J. (2019). Faecal microbiota dynamics and their relation to disease course in Crohn’s disease. J. Crohn’s Colitis.

[55] Öhman L., Lasson A., Strömbeck A., Isaksson S., Hesselmar M., Simrén M., Strid H., Magnusson M.K. (2021). Fecal microbiota dynamics during disease activity and remission in newly diagnosed and established ulcerative colitis. Sci. Rep..

[56] Braun T., Di Segni A., Benshoshan M., Neuman S., Levhar N., Bubis M., Picard O., Sosnovski K., Efroni G., Farage Barhom S. (2019). Individualized dynamics in the gut microbiota precede Crohn’s disease flares. Am. J. Gastroenterol..

[57] Guo X., Huang C., Xu J., Xu H., Liu L., Zhao H., Wang J., Huang W., Peng W., Chen Y. (2021). Gut microbiota is a potential biomarker in inflammatory bowel disease. Front. Nutr..

[58] Cui L., Morris A., Ghedin E. (2013). The human mycobiome in health and disease. Genome Med..

[59] Chin V.K., Yong V.C., Chong P.P., Amin Nordin S., Basir R., Abdullah M. (2020). Mycobiome in the gut: A multiperspective review. Mediat. Inflamm..

[60] Limon J.J., Tang J., Li D., Wolf A.J., Michelsen K.S., Funari V., Gargus M., Nguyen C., Sharma P., Maymi V.I. (2019). Malassezia is associated with Crohn’s disease and exacerbates colitis in mouse models. Cell Host Microbe.

