# Supplementary material for: Evaluation of Bacterial and Fungal Biomarkers for Differentiation and Prognosis of Patients with Inflammatory Bowel Disease
Source: Microorganisms. 2023 Nov 29;11(12):2882. doi: 10.3390/microorganisms11122882 (PMC10745905; doi:10.3390/microorganisms11122882)
Supplement: Supplementary file 1 [file microorganisms-11-02882-s001.zip › microorganisms-2724915-supplementary.pdf]

Supplementary table 1. Baseline characteristics of patients with inflammatory bowel disease and the control group

Variables	IBD patients, N (%)	Control group, N (%)
Age (years)	38 (17−78)*	36 (15-71)*
Male sex	77 (77.0)	74 (76.3)
Current smoking	11 (11.0)	9 (9.3)
Charlson comorbidity index[†]	0.17 (0.49)	0
Disease duration (years)	1 (0−23)*	
UC	67 (67.0)	
UC, Disease extent		
Proctitis	17 (25.4)	
Left-sided colitis	22 (32.8)	
Extensive colitis	28 (41.8)	
UC, Partial Mayo clinic score	2 (0-8)*	
CD, Age		
A1 (< 17 years)	4 (12.1)	
A2 (17–40 years)	26 (78.8)	
A3 (> 40 years)	3 (9.1)	
CD, Disease location		
L1 (Ileum)	9 (27.3)	
L2 (Colon)	0 (0.0)	
L3 (Ileocolonic)	24 (72.7)	
CD, Disease behavior		
B1 (Inflammatory)	15 (45.5)	
B2 (Stricturing)	8 (24.2)	
B3 (Penetrating)	10 (30.3)	
CD, CDAI	45 (0-265)*	
History of abdominal surgery	4 (4.0)	
Exposed medications		
Steroids	54 (54.0)	
Immune modulators	40 (40.0)	
biologics	13 (13.0)	
CRP (> 0.5 mg/dL)	16 (16.0)	0 (0)

FCP (> 250 mg/kg)	49 (49.0)

*Median (range)

†Thirteen patients had comorbidities. Details are as follows: 1 myocardial infarction; 1 cerebrovascular disease; 1 dementia, 1 chronic obstructive pulmonary disease, 2 diabetes mellitus, 2 solid tumor (history of breast cancer and thyroid cancer), 7 liver disease

CD, Crohn’s disease; CDAI, Crohn’s disease activity index; CRP, C-reactive protein; FCP, fecal calprotectin; IBD, inflammatory bowel disease; UC, ulcerative colitis.

Supplementary table 2. Taxa showing a significant difference in the abundance in the stool microbiome between patients with inflammatory bowel disease and healthy control (species level)

Taxon name	LDA effect size*	FDR adjusted *p*-value
Species more abundant in IBD than in controls		
Bacteroides vulgatus	4.414	0.011
Escherichia coli group	3.862	0.031
Ruminococcus gnavus	3.846	0.027
JH815484_s	3.600	< 0.001
Lactobacillus ruminis	3.436	< 0.001
Clostridium innocuum group	3.361	< 0.001
Bacteroides_uc	3.293	< 0.001
Anaerostipes caccae group	3.052	< 0.001
Proteus mirabilis	3.048	0.036
Fusobacterium varium group	3.036	0.004
Species less abundant in IBD than in controls		
Ruminococcus bromii	3.965	< 0.001
Fusicatenibacter saccharivorans	3.936	< 0.001
Romboutsia timonensis	3.934	< 0.001
Bifidobacterium catenulatum group	3.911	0.001
Gemmiger formicilis group	3.895	< 0.001
Bifidobacterium adolescentis group	3.812	0.004

Dorea longicatena	3.806	< 0.001
LN913006_s group	3.804	< 0.001
Collinsella aerofaciens group	3.759	< 0.001
Ruminococcus faecis	3.731	< 0.001
Akkermansia muciniphila	3.719	< 0.001
Holdemanella biformis	3.717	< 0.001
Bifidobacterium longum group	3.601	< 0.001
PAC001173_s	3.599	< 0.001
Clostridium celatum group	3.582	0.001
LT907848_s	3.562	< 0.001
Blautia obeum	3.509	< 0.001
Prevotella copri	3.486	0.009
CCMM_s	3.473	< 0.001
Coprococcus comes group	3.460	< 0.001
PAC001051_s	3.435	0.002
Turicibacter sanguinis	3.373	< 0.001
PAC001129_s	3.353	< 0.001
Streptococcus salivarius group	3.333	0.024
Eubacterium hallii	3.310	0.002
PAC001292_s	3.289	0.024
Agathobacter rectalis	3.287	< 0.001
PAC001256_s	3.245	0.020

Pseudomonas veronii group	3.239	< 0.001
PAC001040_s	3.237	0.004
PAC001136_s	3.235	< 0.001
PAC001054_s	3.229	< 0.001
PAC001048_s group	3.171	< 0.001
PAC001430_s	3.115	0.009
Dorea formicigenerans	3.090	< 0.001
PAC001135_s	3.060	< 0.001
PAC001175_s	3.024	< 0.001
Agathobaculum butyriciproducens group	3.004	0.001

FDR, false discovery rate; IBD, inflammatory bowel disease; LDA, linear discriminant analysis. *Only taxa showing LDA effect size ≥ 3.0 were presented.

Supplementary table 3. Functional modules showing a significant difference in expression in the stool microbiome between patients with inflammatory bowel disease and healthy controls

KEGG Module	Definition	LDA effect size	FDR adjusted *p*-value
Increased in IBD compared to the control			
M00647	Multidrug resistance, efflux pump AcrAB-TolC/SmeDEF	3.322	< 0.001
M00646	Multidrug resistance, efflux pump AcrAD-TolC	3.134	< 0.001
M00718	Multidrug resistance, efflux pump MexAB-OprM	3.098	< 0.001
M00060	Lipopolysaccharide biosynthesis, KDO2-lipid A	3.071	< 0.001
M00144	NADH:quinone oxidoreductase, prokaryotes	3.003	< 0.001
M00079	Keratan sulfate degradation	2.982	< 0.001
M00821	Multidrug resistance, efflux pump TriABC-TolC	2.979	< 0.001
M00720	Multidrug resistance, efflux pump VexEF-TolC	2.967	< 0.001
M00011	Citrate cycle, second carbon oxidation, 2-oxoglutarate → oxaloacetate	2.964	< 0.001
M00339	RaxAB-RaxC type I secretion system	2.952	< 0.001
M00326	RTX toxin transport system	2.951	< 0.001

M00571	AlgE-type Mannuronan C-5-Epimerase transport system	2.950	< 0.001
M00696	Multidrug resistance, efflux pump AcrEF-TolC	2.949	< 0.001
M00325	alpha-Hemolysin/cyclolysin transport system	2.947	< 0.001
M00575	Pertussis pathogenicity signature, T1SS	2.943	< 0.001
M00620	Incomplete reductive citrate cycle, acetyl-CoA → oxoglutarate	2.938	< 0.001
M00699	Multidrug resistance, efflux pump AmeABC	2.933	< 0.001
M00697	Multidrug resistance, efflux pump MdtEF-TolC	2.932	< 0.001
M00173	Reductive citrate cycle (Arnon-Buchanan cycle)	2.930	< 0.001
M00709	Macrolide resistance, MacAB-TolC transporter	2.886	< 0.001
Decreased in IBD compared to the control			
M00207	Putative multiple sugar transport system	3.073	< 0.001
M00333	Type IV secretion system	3.061	< 0.001
M00582	Energy-coupling factor transport system	3.043	< 0.001
M00196	Raffinose/stachyose/melibiose transport system	3.000	< 0.001
M00239	Peptides/nickel transport system	3.000	< 0.001

M00237	Branched-chain amino acid transport system	2.952	< 0.001
M00236	Putative polar amino acid transport system	2.940	< 0.001
M00177	Ribosome, eukaryotes	2.903	< 0.001
M00157	F-type ATPase, prokaryotes and chloroplasts	2.882	< 0.001

FDR, false discovery rate; IBD, inflammatory bowel disease; KEGG, Kyoto Encyclopedia of Genes and Genomes; LDA, linear discriminant analysis.

Supplementary table 4. Univariable and multivariable analyses of predictors associated with relapse in patients with inflammatory bowel disease

	Univariable analysis		Multivariable analysis	
Variables	HR (95% CI)	*P*-value	HR (95% CI)	*P*-value
Age (> 40 years)	1.192 (0.699-2.032)	0.519		
Sex (Female)	0.586 (0.287-1.197)	0.142	0.718 (0.344-1.501)	0.379
Current smoker	0.841 (0.559-1.264)	0.404		
Disease duration (>2 years)	1.243 (0.729-2.118)	0.425		
Enterotype based on stool microbiome				
Type 1	Reference			
Type 2	0.945 (0.475-1.878)	0.871	0.773 (0.379–1.574)	0.478
CRP (≥ 0.5 mg/dL)	1.713 (0.859-3.416)	0.127	1.121 (0.528-2.379)	0.767
FCP (≥ 250 mg/kg)	2.156 (1.264-3.677)	0.005	2.111 (1.177-3.785)	**0.012**
Exposed medication				

Variables	Univariable analysis		Multivariable analysis	
	HR (95% CI)	*P*-value	HR (95% CI)	*P*-value
Steroids	1.710 (0.983-2.977)	0.058	1.601 (0.804-3.220)	0.179
Immune modulators	1.491 (0.879-2.527)	0.138	0.962 (0.495-1.871)	0.910
TNF-alpha inhibitors	0.816 (0.385-1.727)	0.595		

P-values were calculated using Cox regression analysis. Relapse was defined as a composite outcome of (i) new use of steroids, immunomodulators, and biologics; (ii) a visit to an emergency department; (iii) hospitalization; or (iv) abdominal surgery. CI, confidence interval; CRP, C-reactive protein; EV, extracellular vesicle; FCP, fecal calprotectin; HR, hazard ratio; TNF, tumor necrosis factor.
